# Identification of the traditional and non-traditional sulfate-reducing bacteria associated with corroded ship hull

**DOI:** 10.1007/s13205-016-0507-6

**Published:** 2016-09-12

**Authors:** Kiana Alasvand Zarasvand, V. Ravishankar Rai

**Affiliations:** Department of Studies in Microbiology, University of Mysore, Mysore, 570006 India

**Keywords:** Phylogeny, SRB, Ship hull, *dsrAB*, Corrosion

## Abstract

Pitting corrosion due to microbial activity is the most severe type of corrosion that occurs in ship hull. Since biogenic sulfide produced by sulfate-reducing bacteria (SRB) is involved in the acceleration of pitting corrosion of marine vessels, so it is important to collect information about SRB community involved in maritime vessel failure. We investigated the SRB community on corroded hull portion of the ship. With the use of common cultural method and 16S rDNA sequencing, ten bacteria with sulfate reduction ability were isolated and identified. They belonged to both traditional (*Desulfovibrio*, *Desulfotomaculum*) and non-traditional (*Citrobacter*) sulfate-reducing bacteria. All the isolates were able to produce a high amount of sulfide. However, only traditional isolates were showing the amplification for the SRB-specific gene, *dsrAB*. Further studies on corrosion potential of these two groups of bacteria showed that in spite of high sulfide and biofilm production by non-traditional SRB, they are less aggressive towards the mild steel compare to the traditional group.

## Introduction

Microbiologically influenced corrosion (MIC) is an electrochemical process, where the presence and activity of microorganisms accelerate the kinetics of corrosion process (Beech and Sunner 2004). MIC is well documented in many industries, including ship industry, offshore oil and gas production, power plants, and coastal industrial plants (Licina and Cubicciotti 1989; Bodtker et al. 2008; Inbakandan et al. 2010).

Maritime vessels are prone to MIC, as they continuously get exposed to seawater which contains a large number of different microbes. The impairment of metal function due to microbial bioactivity leads to substantial economical loss for ship industries (Schultz et al. 2011). The corrosion of ship hulls increases the surface roughness which consequently enhances the frictional drag. This reduces the ship speed which could be compensated by higher fuel consumption to maintain the required speed. Currently, antifouling paint (paints containing biocides) is being employed to prevent microbial corrosion on ship (Almeida et al. 2007). However, the effective action of antifouling paint depends on many factors, such as temperature, salinity and acidity of water, type of metal, and type of microorganism.

Microbial community on ship hull consists of rich and functionally diverse group of bacteria (Inbakandan et al. 2010). Aerobic and anaerobic bacteria, fungi, and yeast are reported to be responsible for the corrosion of ship hull (Wade et al. 2011). Though many bacteria have been isolated from ship hull, information regarding sulfate-reducing bacteria (SRB) communities from hull of ship has not been recorded so far.

SRB are the most troublesome groups of microorganisms involved in MIC in shipping industry (Tsinker 2004). Hydrogen sulfide, an end product of SRB metabolism, reacts with metal ion to form less soluble metal sulfide which later gets deposited on the metal surface and accelerates the process of pitting corrosion. Pitting corrosion reduces hull thickness which consequently affects the strength performance of the hull structure and results in other forms of failures (Jakubowski 2011). Therefore, due to the importance of this group of bacteria in MIC, in the present study, an attempt was made to isolate the sulfate-reducing bacteria from the biofilm formed on the corroded ship hull.

## Materials and methods

### Sample collection

Total five metal pieces with typical signs of corrosion (metal with pits and black deposits) caused by the activity of SRB was harvested from the underwater portion of corroded hull of fishing vessel in Goa, India, as this part is the most vulnerable part of ship for corrosion. Samples were collected from five different depths (sample number one which was the uppermost sample taken from below the waterline and sample number five was the lowermost one) when a ship was in dry-docking. Each sample was washed three times with sterile phosphate buffer solution to remove any loosely attached bacteria. Sessile bacteria were scraped off from each metal piece and transferred to 50 ml sterile glass container filled with enrichment medium. Then, bottles were transferred to the laboratory and incubated anaerobically at 25 °C for 1 week. The growth of SRB was indicated by the turbidity of culture medium and the production of H_2_S. The Postgate’s B medium was used as enrichment medium which contained 5 ml 70 % sodium lactate, 0.5 g KH_2_PO_4_, 1.0 g NH_4_Cl, 1.0 g Na_2_SO_4_, 2.0 g MgSO_4_·7H_2_O, 1.0 g yeast extract, 0.1 g CaCl_2_·2H_2_O, 0.5 g FeSO_4_·7H_2_O, and 20 g NaCl in 1 l of distilled water (pH 7.5–8.0). Sodium thioglycolate (0.1 g) and vitamin C (0.1 g) were added to the media as reducing agents. Resazurin (1 mg/l) also was added to notify the anoxic condition.

### Isolation procedures

The isolation of bacteria from enriched sample was carried out by the serial dilution of enrichment culture (1 ml) in deaerated water. Hundred microliter aliquot from 10^−4^ to 10^−6^ dilutions were spread uniformly over the Postgate’s B agar plate. The plates were incubated in the dark at 25 °C under anaerobic conditions until black colored colonies appeared on the plates (7–10 days). Anaerobic condition generated in an anaerobic jar with Anaerogas Pack (HiMedia, India). The development of anaerobic condition was confirmed using Anaero indicator (HiMedia, India). Later, well-separated black colonies were picked up randomly and streaked on new plates until getting pure bacterial culture. The purity of the culture was checked with microscopic observation.

### DNA extraction and PCR amplification

The genomic DNA was extracted from a single colony of bacteria using the HiPurA bacterial genomic DNA purification kit (HiMedia, India*).* 16S rDNA locus was amplified by universal primer pairs, 27F and 1492 R (Lane 1991). The PCR condition included an initial denaturation of 94 °C for 7 min followed by 35 cycles of 94 °C for 1 min, 56 °C for 1 min, and 72 °C for 1 min with a final extension at 72 °C for 7 min. The amplification of ∼1.9-kb fragment of the dissimilatory sulfite reductase gene (*dsrAB)* also was carried out using DSR1-F and DSR4-R primers (Wagner et al. 1998). The thermal cycling conditions for amplification were an initial denaturation step (7 min, 94 °C) followed by 30 cycles of denaturation (45 s, 94 °C), annealing (45 s, 57 °C), and extension (1 min, 72 °C) and one terminal extension step (7 min, 72 °C). In amplification of *dsrAB* gene, a negative control reaction with *Escherichia coli* (MTCC 40) DNA was included.

### Sequence analysis and phylogenetic analyses

Sequencing of the amplified products was carried out by Sanger’s dideoxynucleotide sequencing method. The sequences were compared with sequences stored in GenBank using the BLAST (basic local alignment search tool) algorithm. Subsequently, a phylogenetic tree was constructed using the neighbor-joining algorithms with the Molecular Evolutionary Genetics Analysis software (MEGA 6).

### Sulfate and sulfide measurement

To measure the amount of hydrogen sulfide produced by bacteria, Cord-Ruwisch (1985) method has been used. After dissolving of precipitated FeS by replacing 5 % of culture media with 4 M HCl, 0.05 ml of bacterial culture was mixed with 1.95 ml of copper reagent [mixture of HCI (50 mM) and CuSO_4_ (5 mM)], and then, OD was measured at 480 nm. The 0.05 ml sterile media mixed with 1.95 ml HCl (50 mM) were used as blank. Later, the result was compared with the standard hydrogen sulfide plot. Sulfate was measured by the BaSO_4_ gravimetric method (Gilcreas 1965), where 20 ml of bacterial culture was mixed with 5 ml of conditioning reagent, and then, the volume has been brought to 100 ml by distilled water. Later, a spoonful of barium chloride crystal was added to each sample, and after, mixing OD was measured at 420 nm. For the control, sterile media have been used. Later, the result was compared with the standard sulfate plot.

### Biofilm formation

To study the biofilm formation of bacteria on metal coupon, first, mild steel coupons (10 mm × 10 mm × 1 mm dimensions) were polished with 240 and 400 grit polishing paper. Then, they were rinsed three times with distilled water, degreased with acetone, and sterilized by immersing in ethanol solution before exposing to the experimental media. Each coupon placed into 50 ml container which was filled with artificial seawater medium (24.6 g NaCl, 0.67 g KCl, 1.36 g CaCl_2_·2H_2_O, 6.29 g MgSO_4_·7H_2_O, 4.66 g MgCl_2_·6H_2_O, 0.18 g NaHCO_3_ in 1 l of distilled water, and pH 7.5–8.0) containing 3 g/l peptone as carbon and energy source. In all the bottles except controls, late-exponential phase culture of bacteria (1 % v/v) has been inoculated. The flasks were incubated at 27 °C in anaerobic condition for 48 h. After incubation, coupons were taken out from the container and rinsed with sterile distilled water to remove the planktonic bacteria and cultural debris. Then, metal coupons were placed in a new screw cap container filled with 10 ml ethanol. After 20 min fixation, ethanol was discarded and metal pieces were air dried and stained for 15 min with 0.1 % crystal violet. Coupons were then rinsed thoroughly, air dried, and then destained using 95 % ethanol. After 30 min incubation in alcohol solution, 100 μl of the dissolved solution were transferred to the wells of 96-well plates, and the optical density (OD) of each well was measured at 570 nm using a microtiter plate reader. Sterile medium was used as control in each condition.

### Corrosion test

To analyse and compare the effect of each isolates on the metal corrosion, weight loss test was carried out. For this test, first, mild steel coupons (50 mm × 20 mm × 1 mm dimensions) were prepared as explained above and placed in artificial medium. Then, each coupon placed into container which was filled with 1 l artificial seawater medium. Later, bacteria inoculated in all the bottles except controls. The flasks were incubated at 25 °C for 15 days, and then, they were sampled. Coupons were scraped using a sterile scalpel to remove bacterial biofilm. For the mass loss test, coupons were cleaned by washing in acid, neutralizing with sodium bicarbonate, rinsing in water and acetone, and drying in an air stream (Nemati et al. 2001). The weight loss was calculated by subtracting the weight of each coupon which was measured by a digital balance before starting the test and after cleaning of each coupon at the end of the incubation period. Later, the corrosion rate of metal coupons with 8.96 g/cm^3^ density was calculated using the following formula (Ghafari et al. 2013):

$$C = (534W/DAT) \times 0.0254,$$

where *C* is the corrosion rate (mm/year), *W* is the mass loss of the coupons (mg), *D* is the density of sample (g/cm^3^), *A* is the area of sample (in.^2^), and *T* is exposure time (h). Medium replenishment was done every 7 days. The experiments have been done in triplicate.

## Result and discussion

Seawater contains the variety of oxidized sulfur compounds with sulfate being the most predominant. Sulfate can be utilized by assimilatory and dissimilatory sulfate-reducing bacteria. While in assimilatory sulfate reduction, small amounts of sulfate are reduced to H_2_S intra-cellularly for the synthesis of cellular material, dissimilatory sulfate reducers utilize sulfate in large scale for the production of energy, and they secrete copious amounts of sulfide from the cell (Batzer and Sharitz 2014).

Dissimilatory sulfate reducers or SRB are one of the well-known bacteria associated with microbiologically influenced corrosion process. Though these bacteria exist extensively in seawater, they only become a problem when they proliferate on the metal of ship hull docked in polluted environment (Enning and Garrelfs 2014). Therefore, it is important to use biocide for controlling SRB growth. Since the selection of appropriate and effective biocide is dependent on the type of organisms, in the present study, biofilm formed on five different zone of corroded ship hull was used for isolation of sulfate-reducing bacteria. After the enrichment and plating of the bacteria on specific SRB media, one representative of morphologically distinct bacterial type from each sample was randomly selected and used for further studies.

### 16S rDNA based identification

**Fig. 1:**
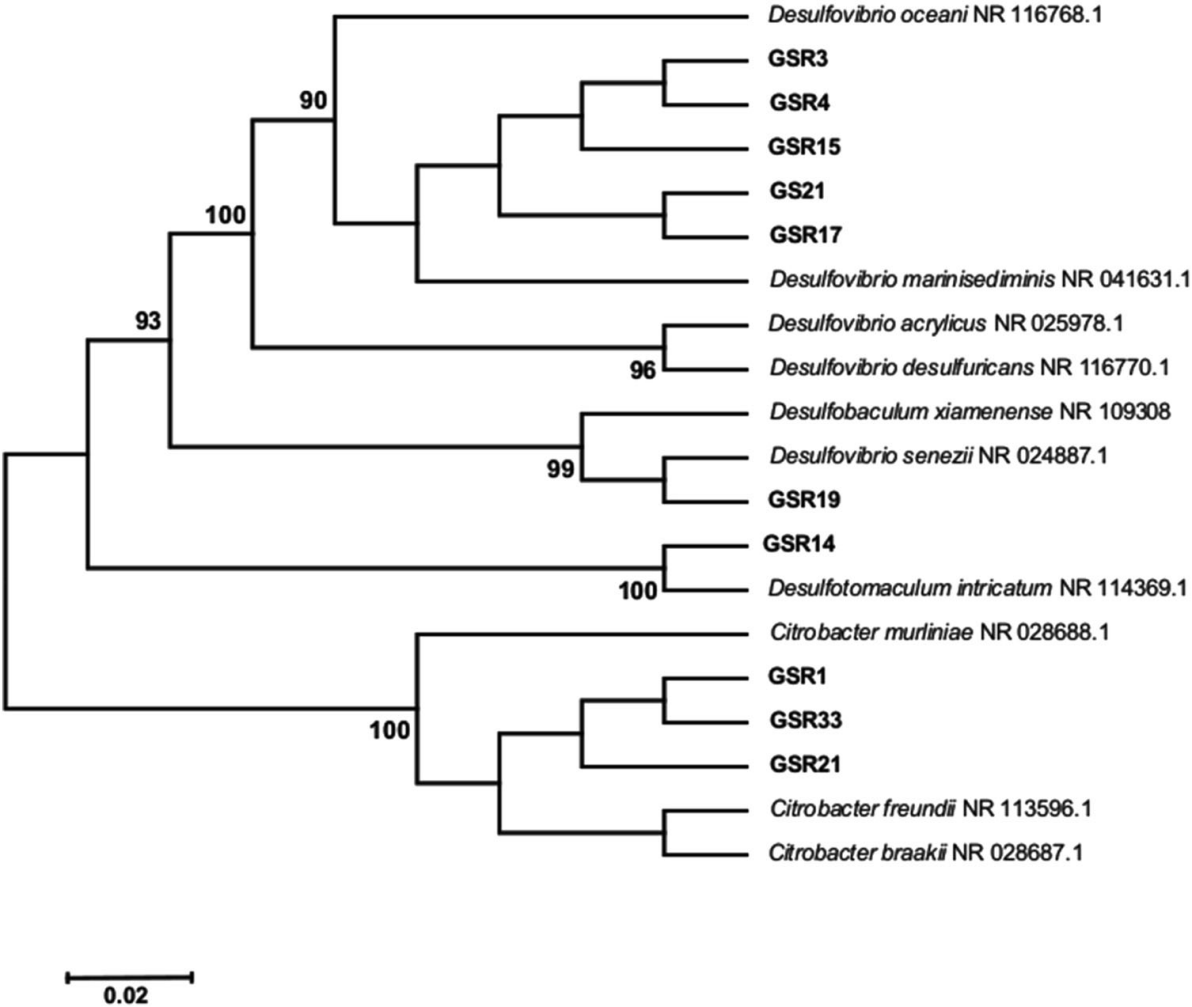
Phylogenetic tree of partial 16S rDNA obtained from corrosive biofilm on ship hull

The composition of SRB communities isolated from corroded samples collected from hull of ship was determined by the sequencing of the 16S rDNA gene. All the ten sequences were submitted to GenBank, NCBI (National Center for Biotechnology Information), and their accession numbers plus their systematic position are presented in Table 1. The sequences which were submitted to BLAST search for retrieving the corresponding phylogenetic relatives showed that all the isolates are having above 97 % similarity to the closely related bacteria. Based on the sequence obtained, a neighbor-joining phylogenetic tree was constructed to visualize the relationship among the isolated strains of the biofilm (Fig. 1). The tree contains two major clades; one consisting of members of common sulfate-reducing bacteria belongs to class Deltaproteobacteria and Clostridia and the other consisting of bacteria belongs to class Gammaproteobacteria.

**Table 1:** Systematic positions of the isolated SRB from biofilm formed on corroded ship hull

Assigned code	Sample no.	Phylum	Class	Order	Genus	Species	GenBank accession no.
GSR1	1	Proteobacteria	Gammaproteobacteria	Enterobacteriales	*Citrobacter*	*freundii*	KT368814
GSR3	3	Proteobacteria	Deltaproteobacteria	Desulfovibrionales	*Deulfovibrio*	*marinisediminis*	KR303707
GSR4	1	Proteobacteria	Deltaproteobacteria	Desulfovibrionales	*Desulfovibrio*	*marinisediminis*	KR349309
GSR 14	5	Firmicutes	Clostridia	Clostridiales	*Desulfotomaculum*	*intricatum*	KT223435
GSR15	2	Proteobacteria	Deltaproteobacteria	Desulfovibrionales	*Desulfovibrio*	*marinisediminis*	KR349310
GSR 17	5	Proteobacteria	Deltaproteobacteria	Desulfovibrionales	*Desulfovibrio*	*marinisediminis*	KT373802
GSR 19	5	Proteobacteria	Deltaproteobacteria	Desulfovibrionales	*Desulfovibrio*	*senezii*	KT373804
GSR21	2	Proteobacteria	Gammaproteobacteria	Enterobacteriales	*Citrobacter*	*freundii*	KT368815
GSR33	3	Proteobacteria	Gammaproteobacteria	Enterobacteriales	*Citrobacter*	*freundii*	KT368816
GS21	4	Proteobacteria	Deltaproteobacteria	Desulfovibrionales	*Desulfovibrio*	*Marinisediminis*	KR094971

Within Deltaproteobacteria, five isolates showed a strong similarity (99.9 %) with *Desulfovibrio marinisediminis* C/L2 (NR 041631) and one isolate showed 99 % similarity with *Dv. senezii. Desulfovibrio* were the most frequent group among isolated SRB (five *Dv. marinisediminis* isolates from five different samples and one *Dv. Senezii* isolate from sample no. 5). This result can be explained by the easy and rapid growth of *Desulfovibrio* which excludes other SRBs while competing for the same substrate. Laanbroek et al. (1984) reported that *Desulfovibrio* have higher affinity for sulfate compared to *Desulfobulbus* and *Desulfobacter* spp. and the former can out-compete the latter when the amount of sulfate available is limited*. Desulfovibrio* are anaerobic cells with high oxygen-tolerance (Sass and Cypionka 2007). This could be another reason for their isolation from the uppermost sample (with the highest oxygen exposure) to lowermost one (with least exposure to the oxygen)*. Desulfovibrio* have been isolated previously from corroded hull of an oil storage vessel and copper piping system of a surface ship (Feio et al. 1998).

Sample no. 5 which was taken from the bottom part of the hull contained one isolate belonged to Clostridia and it showed 100 % similarity to *Dt. intricatum* SR45. Though bacteria belonging to *Desulfotomaculum* are commonly isolated from freshwater or habitats with relatively low salt concentration, there are reports regarding their isolation from marine environment (Isaksen et al. 1994; Nilsen et al. 1996). *Desulfotomaculum* spp. were also isolated from the industrial environment, such as cooling tower and crude oil field (Anandkumar et al. 2009; Cetin and Aksu 2009).

The class Gammaproteobacteria had three isolates which were belonging to the Enterobacteriales order and all were showing high degree of similarity (99–100 %) to *Citrobacter freundii*. There are several reports from the isolation of these bacteria from corroded samples along with SRB bacteria (Neria-Gonzalez et al. 2006; Bermont-Bouis et al. 2007; Agrawal et al. 2010). The isolated Citrobacter was present in sample 1–3; samples which were exposed to higher oxygen concentration. *Citrobacter* is facultative anaerobic bacteria, and they are associated with Enterobacteriaceae family. Sulfide production is wide spread among Enterobacteriaceae. However, *Citrobacter* is the only organism which can reduce sulfate. Angeles-Ch et al. (2002) isolated a strain of *Citrobacter amalonaticus* from the corroded gas pipeline. They mentioned that this strain of *Citrobacter* is able to reduce sulfate to sulfide. The isolation of bacteria belongs to *C. freundii* with sulfate reduction capability shows that the diversity of SRB still has the possibility to be expanded (Zhang et al. 2015; Zhou et al. 2015).

### Sulfate reduction and sulfide production

**Fig. 2:**
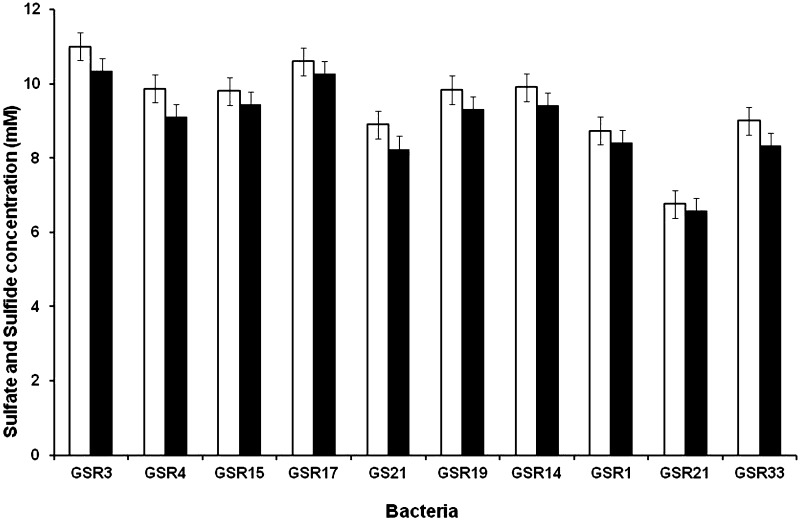
Utilization of sulfate (*white*) and production of hydrogen sulfide (*black*) by isolated bacteria

Though the cultural and molecular results indicating the presence of *Citrobacter* spp. with sulfate reduction ability, to ensure the accuracy of the results, more experiments are carried out by emphasizing on hallmark characteristic of dissimilatory sulfate-reducing bacteria (i.e., consumption of high amount of sulfate and production of copious amount of sulfide). Figure 2 shows the amount of sulfate reduced and sulfide produced by each isolates. As expected high sulfate reduction was the character seen in all the traditional SRB. Strain GSR1, GSR21, and GSR33 reduced 8.5, 6.2, and 9.7 mM of sulfate, respectively.

Bacteria produced 7–11 mM sulfide. The highest concentration of sulfide (11 mM) has been produced by *Dv. marinisedimins* GSR3 and GSR17, and the least amount of sulfide (7 mM) was produced by *C. frundii* GSR21. Sulfide produced by all the three non-traditional SRB was less compared to the amount of sulfide produced by the traditional SRB. However, the amount of sulfide produced in the test was sufficient to consider these strains of *Citrobacter* as sulfate-reducing bacteria.

Zhou et al. (2015) have reported the isolation of *C. frundii* from the sludge of a paper mill located in Tianjin, China. This bacterium reduced 12 mM of sulfate within 7 days. In another study, Qiu et al. (2009) isolated another species of *Citrobacter* (with 98 % similar to *C. freundii*, *C. braakii,* and *C. werkmanii*) from mining area with good sulfate reduction potential (this strain produced 4 mM sulfide). Due to the production of high amount of sulfide, they suggested that sulfate reduction by this bacterium could be through dissimilatory pathway.

### Amplification of *dsrAB* gene

Since the isolated *Citrobacter* was able to produce hydrogen sulfide, while sulfate was the only sulfur compound in the media, we tried to find out whether *Citrobacter* are also reducing sulfate through dissimilatory pathway by checking the presence of *dsrAB* gene.

**Fig. 3:**
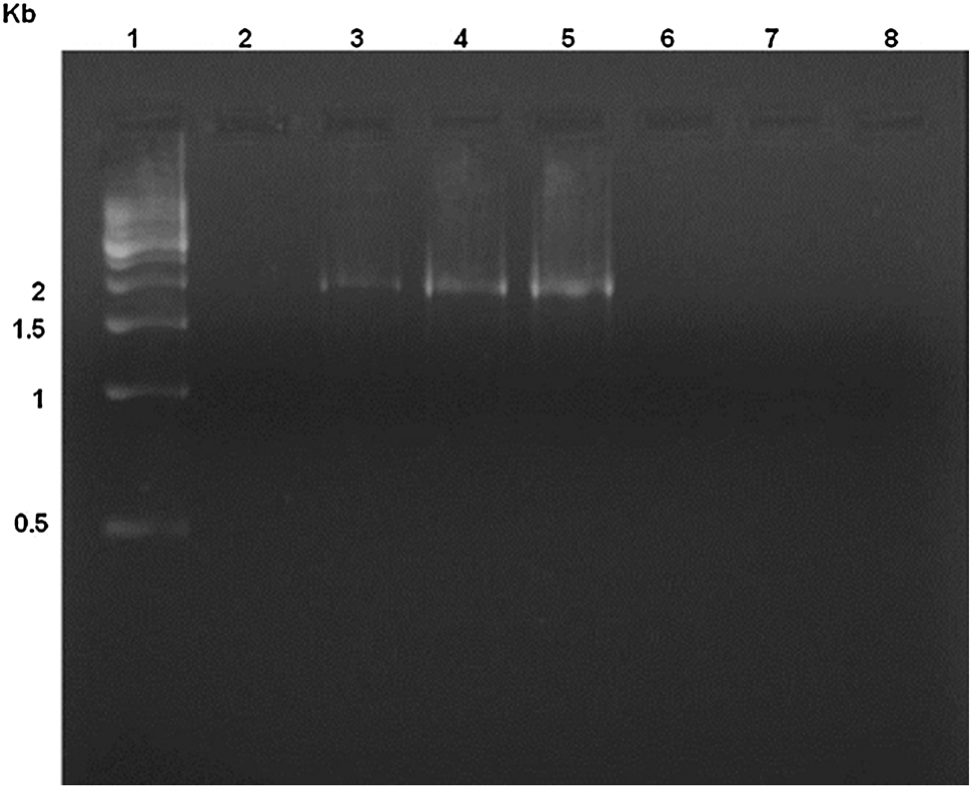
Amplification of *dsrAB* gene using DSR1F-DSR4R with genomic DNA from *Citrobacter freundii* GSR1 (*lane 2*), *Desulfovibrio marinisediminis* GSR3 (*lane 3*), *Desulfovibrio senezii* GSR19 (*lane 4*), *Desulfotomaculum intricatum* GSR14 (*lane 5*), *Citrobacter freundii* GSR21 and GSR33 (*lanes 6* and *7*), and *Escherichia coli* MTCC40 (*lane 8*). *Lane 1* represents the 500 bp DNA ladder

*dsrAB* gene encodes the dissimilatory sulfite reductase, an enzyme that catalyzes the six-electron reduction of sulfite to sulfide during anaerobic respiration (Wagner et al. 1998). Wagner et al. (1998) demonstrated that primer pairs DSR1 and DSR4 are specific for the *dsrAB* gene encoding enzyme which has the ability to reduce sulfate. Using *dsrAB* marker gene, bacteria have been divided into two groups; those containing *dsrAB* gene (or traditional SRB) (*Dv. marinisediminis* strain GSR3, GSR4, GSR15, GS21, GSR14, GSR17,GSR20, *Dv. senezii* GSR19, and also *Dt. intricatum* strain GSR14) and others which did not contain the same gene (or non-traditional SRB) (*C. freundii* strain GSR1, GSR21, and GSR33) (Fig. 3). Yang et al. (2010) isolated a sulfate-reducing *Citrobacter* sp. (Strain SR3) which contains dissimilatory sulphite reductase gene. However, all the three isolated *Citrobacter* in our study were lacking these genes which indicate that the isolated *Citrobacter* spp. may have some other strategies for sulfate reduction.

### Biofilm formation on metal

**Fig. 4:**
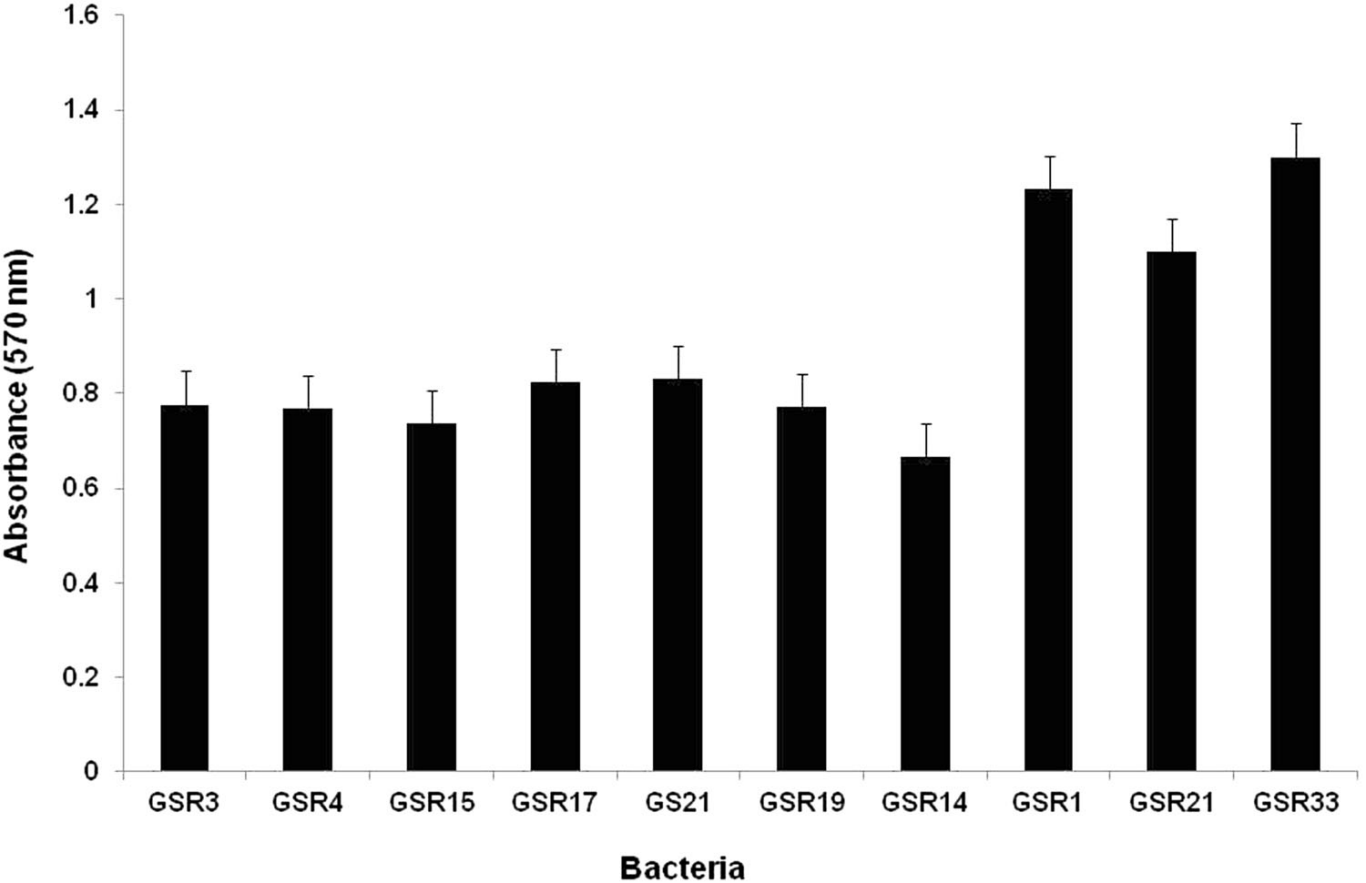
Biofilm forming ability of isolated bacteria after 48 h incubation in anaerobic condition

**Fig. 5:**
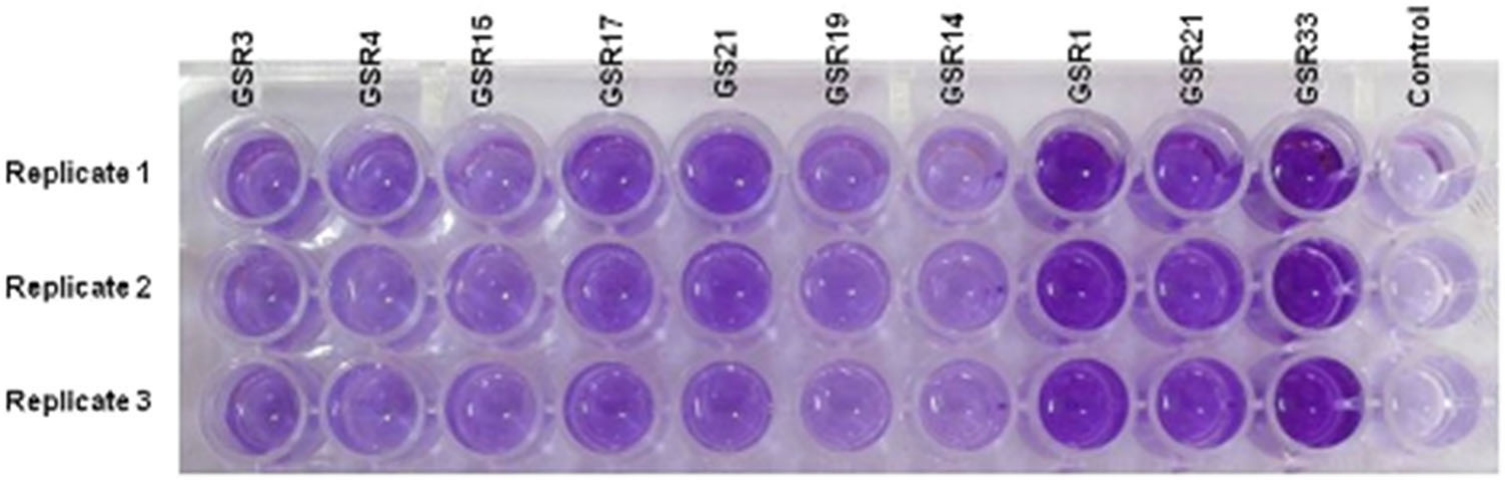
Formation of biofilm by the isolated bacteria differentiated by crystal violate staining in 96 well plates (well No. 1–10 shows the biofilm formation by following strain; GSR3, GSR4, GSR15, GSR17, GS21, GSR19, GSR14, GSR1, GSR21, and GSR33. Well no. 11 is *negative* control)

After observing the sulfate reduction ability of isolated *Citrobacter* spp., we investigated the corrosion potential of them in comparison with the traditional SRB. Since the effect of bacteria on corrosion depends on both biofilm formation ability and their metabolic byproduct, we first examined their biofilm forming ability. The result of this experiment showed stronger biofilm formation in Gram-negative isolates (*Desulfovibrio* spp. and *Citrobacter* spp.) compared to the Gram-positive *Dt. intricatum* (Figs. 4, 5). The difference between the biofilm formations of the bacteria could be explained by the chemical composition of their cell wall. In general, Gram-negative bacteria contain more anionic group in their cell wall which help in the binding of them to the cationic metal surface (Li and Logan 2004). Among the Gram-negative bacteria, *Citrobacter* spp. produced stronger biofilm compared to the *Desulfovibrio* spp. The members of Enterobacteriaceae family are well known as common colonizers on metal surfaces (Bermont-Bouis et al. 2007). In the early stage of biofilm formation, they produce extracellular polymeric substances which facilitate the attachment of other microorganism (Jan-Roblero et al. 2004; Neria-Gonzalez et al. 2006; Bermont-Bouis et al. 2007).

### Corrosion rate

After studying the sulfide production and biofilm formation of each isolate, the influence of these bacteria on metal corrosion was also studied by the mass loss method. In this test, all ten isolates showed a significant weight loss in mild steel compared to the control (Table 2). Corrosion caused by the traditional SRB was higher than the non-traditional one. *Dv*. *marinisediminis* GSR3 which produced the highest hydrogen sulfide showed the highest mass loss (0.3837 mm/year), and the lowest mass loss was observed in *C. freundii* GSR21 (0.1207 mm/year).

**Table 2:** Corrosion rates of mild steel coupons exposed to different isolates

Bacteria	*Dv. marinisediminis*					*Dv. senezii*	*Dt. intricatum*	*C. freundii*			Control
	GSR3	GSR4	GSR15	GSR17	GS21	GSR19	GSR14	GSR1	GSR21	GSR33	
Corrosion rate (mm/year)	0.3837	0.37451	0.3654	0.3759	0.379	0.2512	0.3735	0.1435	0.1207	0.1434	0.0014

In the conclusion, using the cultural method, we isolated ten bacteria, in which three were having unusual sulfate metabolism. The chance of finding species with a novel metabolic activity among hull fouling bacteria is high because of their diverse microbial habitat. They are exposed to a wide range of environment from oceanic waters to contaminated harbor. Therefore, their survival requires constant adaptations to various conditions which lead to the development of multiple pathways (Fu et al. 2004). Though the three isolated *Citrobacter* spp. were less aggressive compared to the traditional SRB, their corrosion potential was highly significant. In view of the fact that the isolated facultative SRB, such as *Citrobacter* spp., have aerobic respiration, and they can multiply rapidly in such an condition, it is important to consider their role while studying MIC, because large numbers of these facultative SRB could create severe MIC under anaerobic condition.

### Nucleotide sequence accession numbers

The accession numbers given to all the ten sequences were submitted to GenBank, NCBI is as follows: KT368814, KR303707, KR349309, KT223435, KR349310, KT373802, KT373804, KT368815, KT368816, and KR094971.
